# Exploring representations of protein structure for automated remote homology detection and mapping of protein structure space

**DOI:** 10.1186/1471-2105-15-S8-S4

**Published:** 2014-07-14

**Authors:** Kevin Molloy, M Jennifer Van, Daniel Barbara, Amarda Shehu

**Affiliations:** 1 Department of Computer Science, George Mason University, 4400 University Drive, 22030 Fairfax, VA, USA; 2 Department of Bioengineering, George Mason University, 4400 University Drive, 22030 Fairfax, VA, USA; 3 School of Systems Biology, George Mason University, 4400 University Drive, 22030 Fairfax, VA, USA

**Keywords:** protein structure space, backbone fragments, remote homologs, Latent Dirichlet Allocation, topic-based representation

## Abstract

**Background:**

Due to rapid sequencing of genomes, there are now millions of deposited protein sequences with no known function. Fast sequence-based comparisons allow detecting close homologs for a protein of interest to transfer functional information from the homologs to the given protein. Sequence-based comparison cannot detect remote homologs, in which evolution has adjusted the sequence while largely preserving structure. Structure-based comparisons can detect remote homologs but most methods for doing so are too expensive to apply at a large scale over structural databases of proteins. Recently, fragment-based structural representations have been proposed that allow fast detection of remote homologs with reasonable accuracy. These representations have also been used to obtain linearly-reducible maps of protein structure space. It has been shown, as additionally supported from analysis in this paper that such maps preserve functional co-localization of the protein structure space.

**Methods:**

Inspired by a recent application of the Latent Dirichlet Allocation (LDA) model for conducting structural comparisons of proteins, we propose higher-order LDA-obtained topic-based representations of protein structures to provide an alternative route for remote homology detection and organization of the protein structure space in few dimensions. Various techniques based on natural language processing are proposed and employed to aid the analysis of topics in the protein structure domain.

**Results:**

We show that a topic-based representation is just as effective as a fragment-based one at automated detection of remote homologs and organization of protein structure space. We conduct a detailed analysis of the information content in the topic-based representation, showing that topics have semantic meaning. The fragment-based and topic-based representations are also shown to allow prediction of superfamily membership.

**Conclusions:**

This work opens exciting venues in designing novel representations to extract information about protein structures, as well as organizing and mining protein structure space with mature text mining tools.

## Background

Genome sequencing efforts utilizing high-throughput technologies are elucidating millions of protein-encoding sequences that currently lack any functional characterization [1,2]. The function of a protein of interest can be inferred from other proteins with a common ancestor, or homologs, with available functional characterization. Either sequence or structure information can be used for this purpose. The majority of methods used for genome-wide functional annotation are based on sequence comparisons and use sequence alignment to identify homologous proteins. Well-known sequence alignment tools include BLAST [3], PROSITE [4,5], and PFAM [6,7]. While typically fast, these tools are restricted to identifying mainly close homologs; that is, pairs of proteins with significant sequence similarity. Function can then be transferred onto an uncharacterized *query *protein when the sequence alignment tool identifies a homolog with known function and no less than 30% sequence identity with the query.

It is often the case that two proteins with similar function cannot be inferred based on sequence information alone. Sequence-based function inference may miss detecting similar proteins where either early branching points (in such case the proteins are referred to as remote homologs) or convergent evolution has resulted in high sequence divergence while largely preserving structure and function. Many sequence-based methods have been offered to extend the applicability of sequence alignment tools for the detection of remote homologs [8-10]. The most successful ones, relying on statistical models learned over multiple aligned sequences, have been shown to improve upon methods based on pairwise sequence comparison but still fail to recognize remote homologs with sequence identity less than 25% [11]. It is worth noting that about 25% of all sequenced proteins are estimated to fall in this category.

The presence of remote homologs was identified as early as 1960, when Perutz and colleagues showed through structural alignment that myoglobin and hemoglobin have similar structures but different sequences [12]. Because structure is under more evolutionary pressure to be preserved than sequence, methods that compare structures allow effectively casting a wider net at detecting related proteins for functional annotation. Structure-based function inference promises to detect remote homologs and expand options for assigning function to novel protein sequences. Many structure similarity methods have been proposed over the years, and two comprehensive comparisons pitching these methods against one another in the context of a gold standard are presented in [13,14]. Well-known methods measuring the similarity of two protein structures include those based on Dynamic Programming (DP) [15-17], including SSAP [18] and STRUCTAL/LSQMAN [19-21], methods based on distance matrices, such as DALI [22], those based on extension of an alignment pinned at aligned fragment pairs or groups of residues, such as CE [23], LGA [24], TMAlign [25], methods based on comparison of secondary structure units, such as VAST [26,27] and SSM [28], and those based on comparison of backbone fragments [29].

Work on effective structure comparison methods has been spurred due to the Structural Genomics Initiative [30] aiming to determine representative structures of all protein families. Such research remains challenging, mainly because the problem of finding the optimal structure similarity score is ill-posed and has no unique answer [31]. While ultimately the purpose is to transfer functional similarity to structurally-similar proteins, it remains open how biologically significant a particular structural alignment is [32,33].

The majority of structure-comparison methods obtain a structure similarity score after aligning the two protein structures provided for comparison. While this is desirable, particularly in cases when the structures need to be analyzed in detail for the locations of high similarity regions, most structure alignment methods tend to be computationally expensive. As such, they are not suitable to be applied at a large scale over structural databases of proteins for the purpose of detecting structural neighbors of a protein of interest. To address this issue, filter approaches have been proposed, where the objective is to rapidly rule out some structures and employ more expensive structure alignment tools on the remaining set of structures.

Most filter approaches for structure comparison rely on finding suitable representations of protein structure so that fast distance measurements can be employed over the representations to rapidly score the similarity of two protein structures without the computationally-intensive step of aligning two structures under comparison [34,29-41]. The representations are typically string or vector-based, and characters or elements are drawn over a pre-compiled alphabet or library of structural features. Representative filter methods include SGM [42], PRIDE [43], and that in [29].

In particular, fragment-based representations of protein structures have been recently proposed to allow fast detection of remote homologs with reasonable accuracy [29]. The representations are based on the bag-of-words (BOW) model of text documents, representing a protein structure as a bag of backbone fragments. Essentially, a representative set of backbone fragments of a given length are compiled over known protein structures [44]. A protein structure of interest is then represented as a vector whose entries record the number of times each of the fragments in the compiled library of fragments approximates a segment in the given protein backbone. The resulting *fragbag *representation has been shown efficient and effective at identifying structural neighbors of a given protein, including close and remote homologs [29]. It is worth noting that fragment-based representations have also been used for structural alignments [45,46].

Due to their efficiency, filter methods are appealing beyond large-scale detection of structural neighbors of a protein query. They can, through the additional application of dimensionality reduction techniques, organize known protein structure space and reveal interesting insight on the relationship between sequence, structure, and function in proteins [34,47,48]. Current applications operate on protein structure space as organized in protein structure databases, such as the "Structural Classification of Proteins" (SCOP) [49] and the "Class, Architecture, Topology, and Homology" (CATH) databases [50,51]. It is worth noting that both databases contain protein domains rather than complete protein structures; that is, these databases break up and organize the known protein structures as deposited in the Protein Data Bank [52] in various ways. Biologists usually break up large proteins that contain multiple unrelated domains spliced together into one polypeptide based on a process that involves analysis of sequence, structure, and domain-specific expertise into what constitutes a domain. Both SCOP and CATH are hierarchical, as opposed to the "Families of Structurally Similar Proteins" (FSSP) database [53]. In SCOP and CATH, domains are first grouped/classified together based on common secondary structure components (this is known as Class), then common arrangement (Architecture in CATH), topology of secondary structure elements (fold in SCOP and Topology in CATH), and then homologous superfamilies (Superfamily in SCOP and Homologous family in CATH) and sequence families (family in both SCOP AND CATH). Unlike SCOP, where the classification is largely manual, CATH is more automated and explicitly uses sequence and structure-based criteria for assigning homology.

The fragbag representation has been recently employed to embed the protein structure space through simple linear dimensionality reduction techniques. The obtained low-dimensional maps are shown to provide interesting insight on the relationship between structure and function in the currently known protein universe [47] organized in SCOP [49] and CATH [51]. Other representations and ensuing maps have been obtained by other researchers over the years, showing, for instance, a closer relationship between structure and function than sequence and function [34]. We confirm some of these findings in this paper, showing that an embedding of the fragbag-based space through Principal Component Analysis (PCA) is low-dimensional and groups structurally-similar domains together.

In this paper, we present work on a novel low-dimensional categorization of the protein structure space. We seek representations that separate classes and capture the unique structural information in a class without relying on posterior dimensionality reduction techniques. We investigate a topic-based representation obtained through application of the Latent Dirichlet Allocation (LDA) model. A topic-based representation of protein structure has been proposed recently in [54] as an alternative to fragbag, but the study has been limited to employment of topics to identify structural neighbors of a given protein. We conduct a detailed analysis of the quality and information captured by topics, building on our previous work on topic-based representations of text documents in text mining [55]. We additionally demonstrate that a topic-based representation is just as descriptive and accurate as the fragment-based one not only at identifying remote homologs but also at organizing protein structure space. In particular, we demonstrate through the use of the ChiSquare significance test that many SCOP superfamilies are statistically significant in the definition of the topics, essentially giving semantic meaning to topics in the same way that a group of text documents gives meaning to and defines a certain topic. Moreover, we show that the fragbag and topic-based representations allow binary classifiers to accurately predict SCOP superfamily membership of protein structures. We believe the work presented in this paper opens exciting venues in designing novel representations to extract information about protein structures, as well as organizing and mining protein structure space with mature text mining tools.

## Methods

We first summarize the fragbag representation of a protein structure, followed by a brief description of PCA. The LDA model is summarized next, with further description of the topic-based representations it offers on proteins and the measurements used to conduct the analysis over topics.

### Fragbag BOW representation of protein structure

The fragbag representation is based on the Kolodny fragment libraries [44] and is based on the concept of a *C*_α_-based molecular fragment. A library of fragments of *l_f _*amino acids in [44] is constructed as follows. Fragments of *C*_α _traces of 200 accurately-determined protein structures are clustered, depositing the representative of each cluster in the fragment library. While analysis on the fragbag representation considers fragment libraries with fragments of length $l_{f}\in \left\{6,...,12\right\}$, we focus on fragments of length 11 in this paper, shown to result in the highest accuracy in identifying structural neighbors in [29,54] and our own analysis (data not shown).

The concept of molecular fragments allows obtaining a vector-based representation of a protein structure as follows. Given a fragment library of *F *fragments of a fixed length *l_f_*, a protein structure *P *can be represented as a vector *V *of F entries. Different information retrieval (IR) techniques can be used to fill an entry *V_i _*associated with fragment *f_i _*in the library(1 ≤ *i *≤ *F*). For instance, entry *V_i _*can record the presence or absence of fragment *f_i _*(stored at position 1 ≤ *i *≤ *F *in the library) in *P*, effectively resulting in a boolean vector. Alternatively, the number of times fragment *f_i _*is found in *P *can be used. This is also known as term frequency (TR) and is the method employed by the *fragbag *representation in [29]. Generally, other naive vector space models can be used, including term frequency-inverse document frequency (TF-IDF) [56].

**Figure 1:**
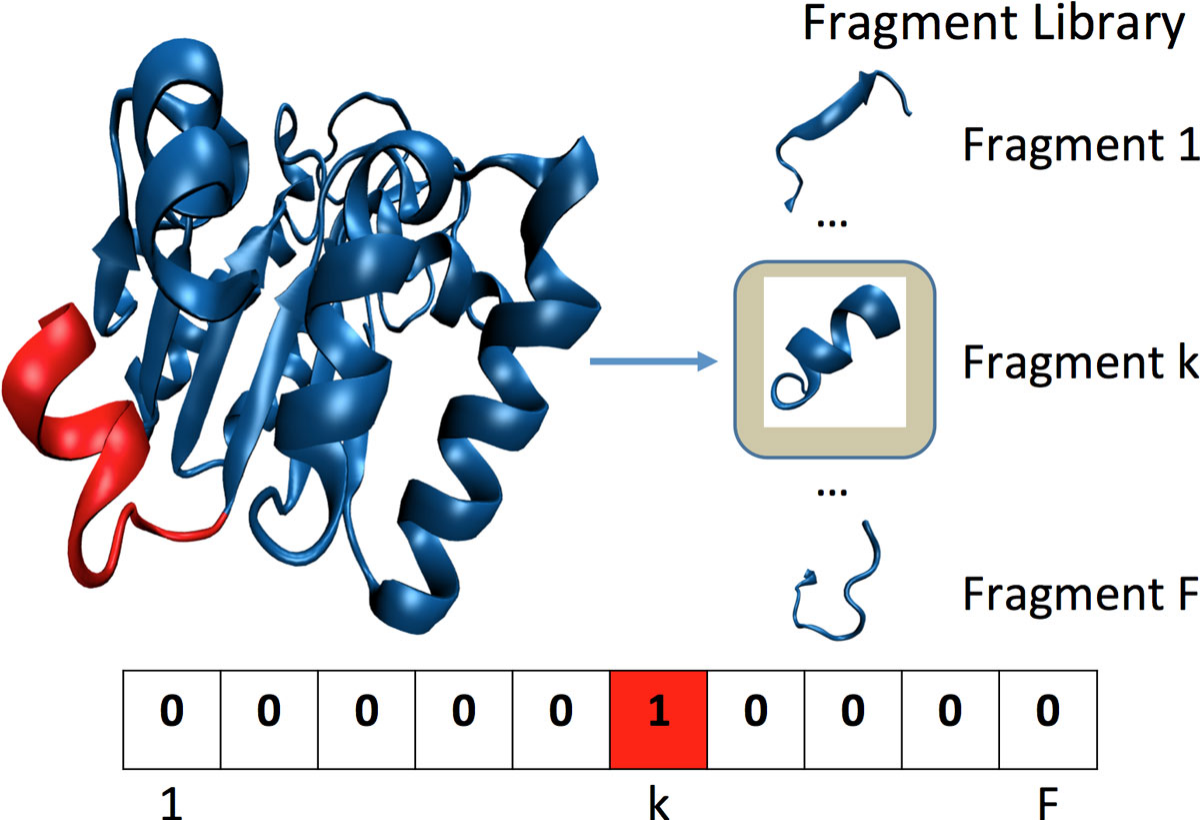
**Molecular fragment replacement process**. A protein structure is shown on the left, rendered with VMD [67] using the NewCartoon graphical representation. The protein structure is scanned one fragment at a time from the N- to the C-terminus. The first fragment is highlighted in red. The position of the fragment in the fragment library is identified, and the entry in the BOW vector at that particular position is incremented. After the entire structure is scanned, the resulting BOW vector is the one supplied to LDA.

The presence of a fragment *f_i _*in *P *is detected as follows. The *C_α _*trace of *P *(that is, only *C_α _*coordinates are extracted from the protein structure) is inspected at every location *j* in blocks of *f* consecutive amino acids, or segments [*j, j *+ *f - *1]. The *C_α _*coordinates of the particular segment under consideration are compared to each fragment f in the library (1 ≤ *i *≤ *F*), and the fragment with the lowest least-root-mean-squared-deviation (lRMSD) is reported as the fragment matching the particular segment (least in lRMSD stands for optimal RMSD after removing deviations due to rigid-body motions, and RMSD is the Euclidean distance weighted over the number of points) [57]. The entire process is illustrated in Figure 1.

Given this representation, any distance or similarity measurements can be used over the fragbag vectors of two protein structures to measure their structural distance or similarity. In [29], various distance measurements are tested, including the basic Euclidean distance as well as cosine distance (which measures the angle between two vectors). The cosine distance is reported to be most accurate and competitive with top structure-alignment methods in detecting structural neighbors.

### Low-dimensional embedding of protein structure space

Given fragbag representations of protein structures, the newly defined (fragbag) vector space, which has dimensionality 400, can be reduced to a few dimensions through various dimensionality reduction techniques. In [47], PCA has been used to project SCOP domains on the two top principal components (PCs). PCA is a well-known linear dimensionality reduction technique, which finds an orthogonal transformation of points given in some original high-dimensional space such that the transformation highlights new axes, also known as the PCs, that maximize variance in the projected or transformed data. Typically, the transformation is said to yield a reduced or low-dimensional embedding when a few, 3-5, PCs retain more than 70% of the variance in the original distribution of the data [58]. We apply PCA here, as well, to visualize co-localization of function in the protein structure space and qualitatively compare these results with the organization readily obtained through the topic-based representation we investigate in this paper.

### LDA-based topic representation of protein structure

We propose an alternative representation of protein structure in this paper based on topics obtained through a popular technique in text mining, LDA. LDA was introduced in [59] as a generative probabilistic model to find latent groups (topics) that capture the structure of observations represented by BOW models, which in this setting are generated using the *fragbag *method. The key idea, first introduced in [54] but limited to detection of structural neighbors, is to represent proteins as probability distributions over latent topics, which are themselves probability distributions over fragments in the fragment library. This idea builds on the original one introduced to categorize text documents of a given corpora by the topics covered in each of them. In text mining, however, visual inspection of the words of highest probability in each topic allows giving semantic meaning to topics. Associating semantic meaning to protein fragments (analogous to words in this setting) is not easy, and we provide in this paper a series of analysis techniques to do so.

**Figure 2:**
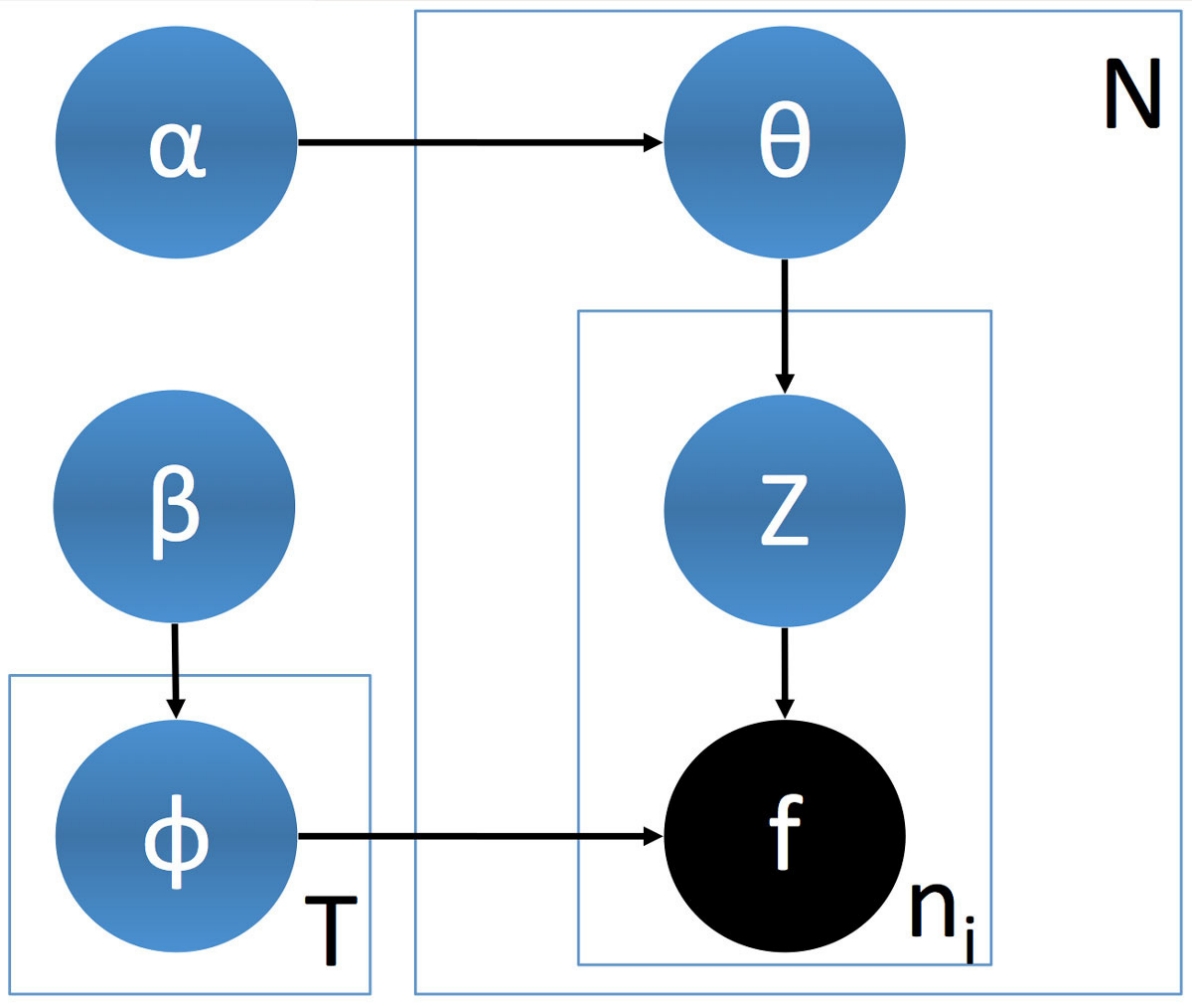
**LDA plate**. *T *is number of topics, *N *is the number of protein structures. Each fragment within a protein is represented by *f *and *n_i _*is the number of fragments in *P_i_*. Blue and black backgrounds indicate latent and observed variables respectively.

We briefly describe the concepts of LDA and how they map to our investigation of proteins. The graphic model for LDA is shown in Figure 2. The generative process in this model functions as follows. First, a multinomial distribution, *ϕ_t_*, is assigned to each topic 1 <= *t *<= *T*. Each of these distributions represents the probability of each fragment in *F *participating in topic *t*. For each protein *P_i _*that is constructed, we obtain a mixture of topics by assigning another multinomial distribution, *θ_i_*. Each fragment in protein *P_i _*is generated by first selecting a topic *t *according to *θ_i_*, and then using that topic's distribution *ϕ_t _*to select the actual fragment. Each fragment within each protein represents a latent variable, *z_i_*, that is assigned to a specific topic. The assignment of multinomial distributions is obtained from a Dirichlet distribution, which is the conjugate prior for the multinomial distribution. As such, each sample from a Dirichlet yields a multinomial distribution. Separate Dirichlet distributions are used for sampling the distribution of topics within a protein (*θ_i_*) and for the distribution of fragments within a topic (*ϕ*_t_) and are parameterized by *α *and *β *respectively.

The goal in LDA is to maximize the likelihood of the posterior through the refinement of the topic assignments *z_i_*. This is accomplished using the LDA algorithm from [60]. This method initially assigns each *z_i _*to a random topic and then utilizes many iterations of Gibbs sampling to approximate the *ϕ *and *θ *distributions. We direct the reader to [60] for a more detailed discussion of LDA and this specific approximation algorithm.

In this context, topics make for general representations of proteins, under which a protein is treated as a mixture of many topics, albeit with different probabilities. As we relate in Results, one can employ these topic-based representations to identify structural neighbors of a protein. We additionally show how topics categorize the protein structure space, revealing interesting insight into what it is that each topic captures about protein structure and function.

#### Evaluating information content in topics

One of the parameters in LDA is the number of topics *T*. Tuning *T *can be accomplished by measuring the information gain provided in each topic compared to a baseline [55]. The distribution of fragments over the entire protein structure space, as available in SCOP, for instance, can be used to represent a baseline distribution over fragments. Each topic obtained by LDA is also a probability distribution over fragments. We use the symmetric Kullback-Leibler (KL) divergence [61] to measure the information gain of each topic over the baseline distribution. Briefly, given two probability distributions *p_0 _*and *p_1_*, $\text{KL}\left(p_{0},p_{1}\right)=\sum p_{0}\left(x\right)\cdot ln\frac{p_{0}\left(x\right)}{p_{1}\left(x\right)}$ We use a symmetric version of KL defined as 0.5 $\left(\text{KL}\left({\text{p}}_{0},{\text{p}}_{1}\right)+\text{KL}\left({\text{p}}_{1},{\text{p}}_{0}\right)\right)$. Larger distances imply higher information gain in each topic as opposed to the baseline distribution of fragments over the entire corpora. Small distances imply that the topic is essentially junk, providing no additional semantic content as compared to the baseline. This evaluation is carried out for each topic in the Results section to additionally measure the information gain as one increases the number of topics requested from LDA.

In addition, log likelihood evaluates how well the data (the fragments defining protein domains) fits the model, which in this case is the topic space model produced by LDA. When performing parameter estimation, a common strategy is to maximize the log likelihood as proposed in [62]. We employ this technique to measure the effectiveness of each LDA model, varying the number of topics *T*. Let *M *represent all the parameters, including *T*, for the LDA model. Equation 1 shows the likelihood of M generating the set of proteins *P*. Taking the log of both sides yields Equation 2. Equation 3 shows the calculation for computing each protein *P_i_*, and taking the log of both sides yields Equation 4. *F *is the total number of fragments used to describe the ensemble and $n_{i}^{\left(v\right)}$ is the number of times fragment *v *appears in protein *P_i_. P*(*f_v_*|*t_k_*) is the probability of the fragment *f_v _*being in topic *t_k_*, which is provided by the multinomial distribution *ϕ_k_. P*(*t_k_*|*P_i_*) is the probability of topic *t_k _*being in protein *P_i_*, which is provided by *θ_i_*. These measurements are shown in the Results section to demonstrate that the log likelihood decreases as the number of topics increases.

(1) $$p\left(P\text{|}M\right)=\overset{N}{\underset{i=1}{\prod }}p\left(P_{i}\text{|}M\right)$$

(2) $$log\;p\left(P\text{|}M\right)=\overset{N}{\underset{i=1}{\sum }}log\;\;p\left(P_{i}\text{|}M\right)$$

(3) $$p\left(P_{i}\text{|}M\right)=\overset{F}{\underset{v=1}{\prod }}{\left(\overset{T}{\underset{k=1}{\sum }}\phi_{k,v}\cdot \theta_{i,k}\right)}^{n_{i}^{\left(v\right)}}$$

(4) $$log\;p\left(Pi\text{|}M\right)=\overset{F}{\underset{v=1}{\sum }}n_{i}^{\left(v\right)}*log\left(\overset{T}{\underset{k=1}{\sum }},(p\left(f_{v}\text{|}t_{k}\right)p\left(t_{k}\text{|}P_{i}\right)\right)$$

### Topic signatures of structural classes and co-localization in protein structure space

Each topic may capture "signatures" associated with different classifications (SCOP, CATH). To test for these signatures, we propose using heatmaps constructed over the LDA-computed topic space. LDA presents the topic space as a *N *x *T *matrix, where *N *is the number of proteins and *T *is the number of topics. The row vector for protein *P_i _*records the number of times a fragment is classified to be within a given topic *T_j_*. Additionally, each protein is assigned a label according to some classification standard; a label corresponds to a class. For instance, a label may be the fold of the protein, as obtained from the top level of the SCOP hierarchy. Alternatively, the label can track the superfamily membership of a protein in SCOP.

Many protein domains are assigned the same label *L_i_*. We sum fragment counts for topic *T_j _*on each protein assigned the same label *L_i_*. This provides us with a fragment count for topic *T_j _*in label *L_i_*. Normalizing over all labels provides us with probability *P*(*L_i_*|*T_j_*). This produces an *L *x *T *matrix, where each column in the matrix sums to one. Results in this paper visualize this matrix as a heatmap, with colors following the low-to-high probabilities in a blue-to-red colors scheme.

When protein classes have strikingly different sizes, the above analysis will be skewed. A high probability *P*(*L_i_*|*T_j_*) may be assigned to a class with label *L_i _*simply because of the high number of domains in the class with label *L_i_*. This situation arises when analyzing topic signatures over the superfamily classification in SCOP. In this case, we take a different approach to obtaining a heatmap that elucidates topic signatures for protein classes. We employ the ChiSquare significance test [63] at a confidence level of 99%. This analysis is performed for each topic *T_j_*. For each protein with label *L_i_*, we compute the number of fragments found within topic *T_j _*(let's refer to this as $C_{T_{j}}^{L_{i}}$), and the number of fragments that are not assigned to proteins with this label ($C_{T_{j}}^{\neg L_{i}}$). We compute these counts for the entire population minus the topic we are currently analyzing ($C_{\neg T_{j}}^{L_{i}}$ and$C_{\neg T_{j}}^{\neg L_{i}}$). These value are used to construct a contingency table and perform the ChiSquare significance test. When the test shows a significant difference, and the population in the topic is greater than the remainder of the population, we characterize this topic as having a signature for the label under consideration.

### Predicting superfamily membership of protein structure

We demonstrate that the fragbag and topic-based representations can be employed by machine learning classification algorithms to predict superfamily membership for a given protein structure. Since this is a multiclass classification problem, we employ the one-vs-all strategy, using 7 binary classifiers, one for each of the 7 most-populated superfamilies in SCOP. We employ the popular Support Vector Machines (SVM) for the binary classifier [64].

The set of 9,852 protein domains in these superfamilies is extracted, and LDA is applied to this set. When using the topic-based representation, each protein's multinomial distribution across the topic space returned by LDA serves as its coordinates in the 10-dimensional space (our analysis in the Results section makes the case that no more than 10 topics are needed). The resulting 10-dimensional vectors are treated as a training dataset, and 7 classifiers are built (SVM is a binary classifier) in order to predict superfamily membership with binary classifiers. When using the fragbag representation, the training vectors are 400-dimensional as opposed to topic vectors which are 10-dimensional.

When building an SVM classifier for superfamily *i *(1 ≤ *i *≤ 7), the set of training vectors corresponding to domains in that superfamily are treated as the positive training dataset. The rest of the vectors, corresponding to domains in other superfamilies are treated as the negative training dataset. We note that for some of the superfamilies, there are many more negative instances than positive ones, as expected. In such cases, re-balancing of data is performed by undersampling the negative class in order to achieve an equal count of positive and negative instances.

Each SVM classifier is trained independently (on each superfamily), using a polynomial kernel and a soft margin parameter *C *= 1.0. Ten-fold cross-validation is used to measure the classification performance, as related in the Results section. For each protein domain, the prediction among the 7 classifiers that has the highest confidence is chosen as the final prediction for that domain. In this way, superfamily membership is predicted for each family, and standard TPR, FPR, and accuracy measurements can be used to evaluate performance.

## Results and discussion

### Implementation details, datasets, and experimental setup

We use a MATLAB implementation for LDA [60]. All our experiments and analysis are executed on a 2.4Ghz Core i7 processor. Parameter values for LDA are *α *= 50/(number of topics) and *β *= 200/(fragment library size). Extracting the fragbag representation for each protein domain in a dataset of 31,155 domains (datasets are detailed below) takes 10 hours. LDA runtimes depend on the number of topics requested and vary from 2 hours for 10 topics to 24 hours for 200 topics. The following analysis conducted here is organized in four sets of experiments. The WEKA data mining package [65] is employed for training SVMs on superfamily-labeled protein structures as described in the Methods section.

We first tune LDA varying the number of topics to show that most information can be obtained with a relatively small number of topics. The topics that allow obtaining comparable results in this context are then analyzed in detail in terms of what fragments they capture. This allows associating "semantic" meaning to topics in terms of the over-represented fragments they contain.

Second, we demonstrate that the representation of a protein domain through LDA-obtained topics, as described in Methods, is just as useful as the fragbag representation to capture structural similarity and report structural neighbors with comparable accuracy. We do so over a database of 2,930 sequence-nonredundant structures, extracted from CATH, as in [29,54]. Each structure in this dataset is treated as a query, and structural neighbors are identified for it over the rest of the dataset. This process is repeated for each structure in the dataset to obtain the average area under the curve of receiver operating characteristic (ROC) curves [66]. We place these results in context, comparing to representative structure alignment and filter methods.

Our third set of experiments concerns how topics can be used to organize protein structure space as compared to the fragbag representation. This analysis is placed in context by first demonstrating the usefulness of the fragbag representation in obtaining a low-dimensional map of the protein structure space through PCA. We restrict our analysis and visualization to two levels in the SCOP hierarchy, class and superfamily. The dataset we employ to demonstrate the co-localization of structurally- and functionally-similar proteins (according to classes in a SCOP hierarchy) consists of 31,155 protein domains extracted from SCOP 1.71 [49]. This dataset is kindly provided to us by R. Kolodny, and our choice of this dataset is so that direct comparisons can be drawn with work by Kolodny and colleagues in [47]. We focus the analysis to top-populated families in the two chosen levels, class and superfamily, in the SCOP hierarchy for clarity. We show that classes have unique topic signatures, which further supports our conclusions that LDA-obtained topics are general and informative representations of protein domains. They can be employed to detect remote homologs and obtain further insight about the organization of the protein structure space.

Our fourth and final set of experiments demonstrates that the topic-based representation captures important information about a protein structure that allows predicting superfamily membership. Binary classifiers are used for this purpose to predict one of the 7 most-populated superfamilies for given protein structures. Our results show that both representations allow standard classifiers to achieve high prediction accuracy, which we believe opens the way towards using simple representations for automated and reliable hierarchic classification of proteins in databases such as SCOP and CATH.

### Less is more: topic space is low-dimensional

**Figure 3:**
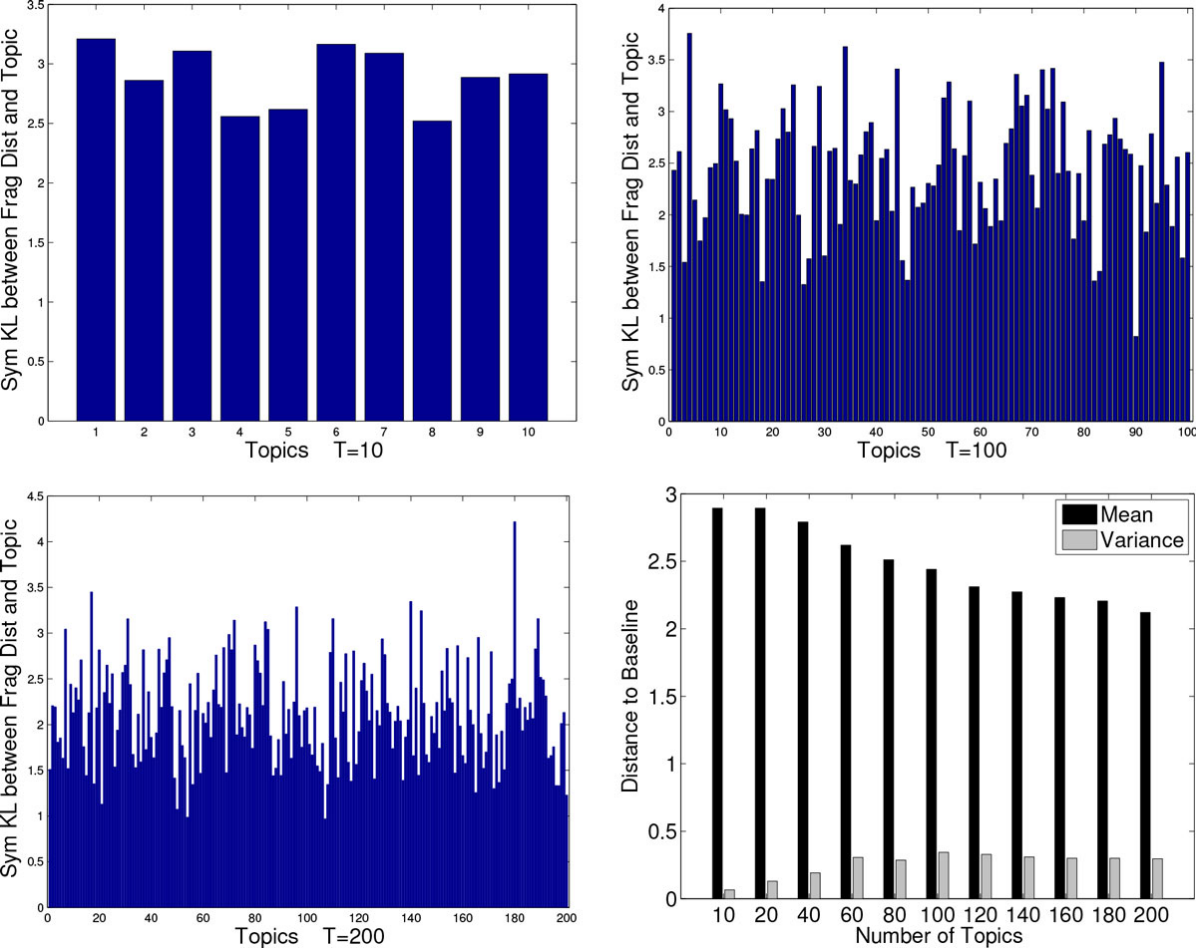
**LDA topic information analysis**. Symmetric KL distance between each topic and the baseline fragment distribution over the entire corpora is shown. Three settings of LDA are compared, where the number of topics varies from 10, to 100, to 200. A quantitative comparison is shown where the number of topics is evaluated at 11 different values.

We show that increasing the number of topics results in topics of low information gain, demonstrating that the chosen number of 10 topics is appropriate. We compute the symmetric KL distance, as described in Methods, to measure the information gain of each topic over the baseline distribution of fragments over all SCOP domains. We do so for 11 different settings of *T*, starting with *T *= 10 through *T *= 200. Figure 3 highlights the value of the KL distances for three settings of *T *(10,100,200). To formulate a quantitative comparison, we compute the mean and variance of each set of KL distances for each of the 11 settings of *T*, which is shown in the bottom right panel of Figure 3. This analysis illustrates that the mean KL distance decreases as the number of topics increases, and the variance increases as the number of topics increase. This suggests that increasing the number of topics does not result in more information and that many topics are essentially "junk" topics for the larger values of *T *[55].

**Figure 4:**
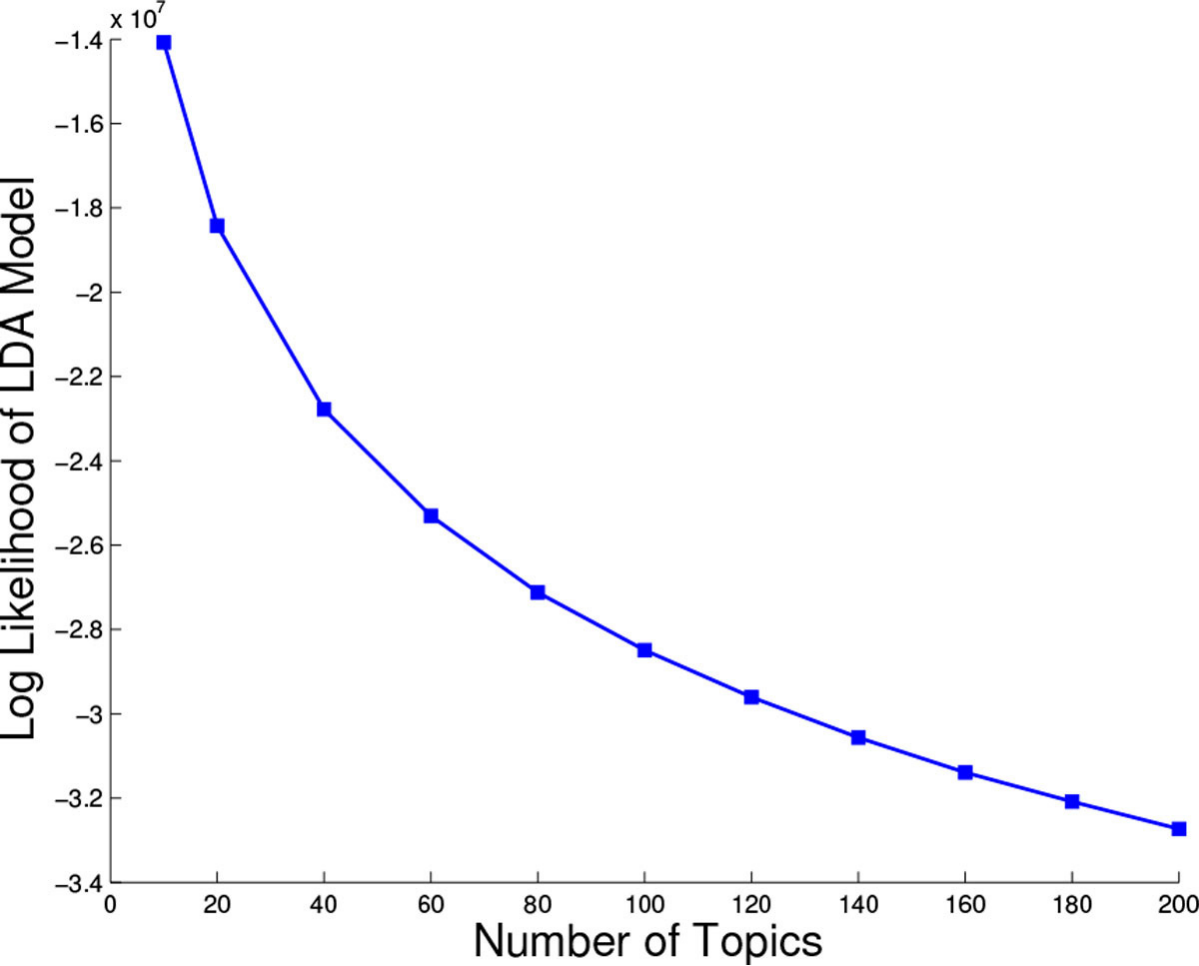
**Topic analysis using log likelihood**. The log likelihood of fitting the data is shown for 11 LDA models, where the number of topics varies from 10 to 200.

Additionally, we show the log likelihood, measured as detailed in the Methods section, for various settings of *T *in Figure 4. As the number of topics increases, the log likelihood decreases. Combining this analysis with that on information gain clearly demonstrates that more topics is not necessarily better. Moreover, these results support the choice of 10 topics as sufficient for the rest of our analysis. It is worth emphasizing that, from now on, a protein structure is represented as a 10-dimensional vector (where each entry in the vector records the probability with which that topic is "found" in the structure). This lies in contrast to the higher-dimensional vector space resulting from the fragbag representation where 400 fragments are employed as opposed to 10 topics. One of the advantages of this lower dimensionality is that dimensionality reduction techniques do not have to be used in order to provide low-dimensional user-friendly embeddings or maps of protein structure space. A component of our analysis below illustrates how topics are signatures of SCOP classes and can even be employed to accurately predict superfamily membership.

**Figure 5:**
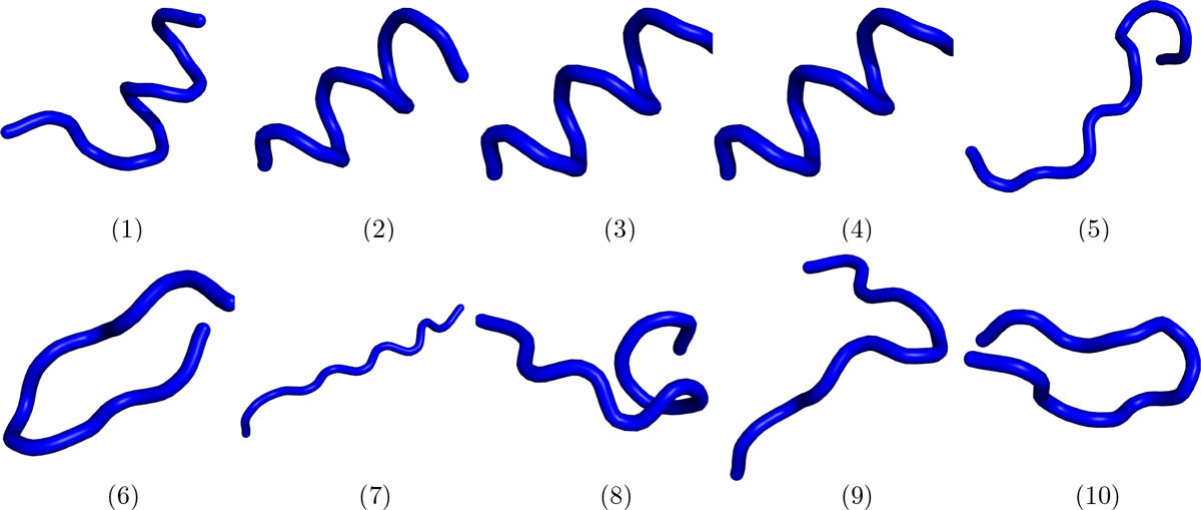
**Top-populated fragment**. The top-populated fragment of each topic is shown.

Before relating results into how the topic-based representation compares to fragbag and other methods in detecting remote homologs and organizing protein structure space, we provide further insight into what the topics capture. In text mining, peeking into the top populated word(s) readily provides semantic meaning into what a topic captures. It is not possible to directly do so in the protein structure space. However, inspecting the top fragment(s) (for lack of space, we limit the visualization to only the top fragment) and correlating this information with analysis on classes most likely to be associated with certain topics provides information into the meaning of a topic in the protein structure space. The top-populated fragments in each topic are shown in Figure 5.

### Detection of close and remote homologs: topics capture structural similarity

We first compare the ability of the topic-based representation vs. fragbag to identify structural neighbors of a protein. We recall that the dataset employed for this analysis is the sequence-nonredundant dataset of 2,930 protein structures extracted from CATH. Each protein in this dataset is treated as a query. The gold standard on which proteins in the dataset are determined to be structural neighbors of a query protein is obtained by a best-of-six structural alignment protocol, courtesy of R. Kolodny. Three different structural alignment scores (SAS) of 5, 3.5, and 2.0Å are employed. A SAS threshold of 2.0Å allows identifying close homologs of a protein, whereas a threshold of 5Å identifies remote homologs. Given a particular SAS threshold and the gold standard of structural neighbors obtained with that threshold, the following experiment is conducted.

Employing the fragbag or topic-based representation and the cosine distance over the particular representation under investigation and continuously varying the decision threshold (that is, the cosine distance between two protein structures under the particular representation), a receiver operating curve (ROC) can be constructed, and the average area under the curve (AUC) score can be reported. The ROC curve plots the true positive rate (TPR = TP/(TP+FN)) vs. the false positive rate (FPR = FP/(FP+TN)) over the decision threshold. Summarizing the ROC with AUC allows associating a score with each query protein. Averaging over all proteins in the dataset, essentially treating each of them in turn as a query protein, allows obtaining an average AUC and thus measuring the effectiveness of a particular representation at capturing structural neighbors. Performing this analysis at the three different SAS thresholds further allows judging the effectiveness at capturing close to remote homologs.

**Figure 6:**
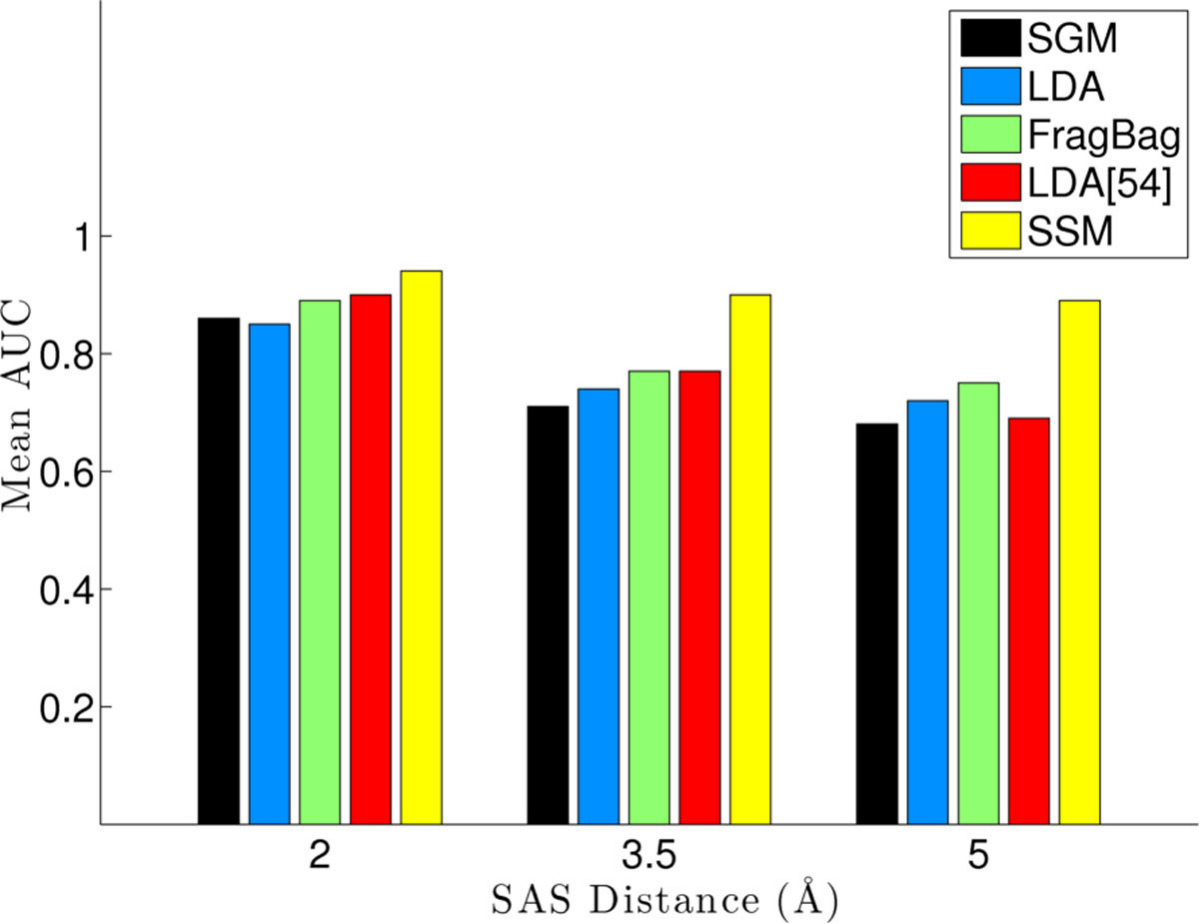
**Average AUCS over SCOP**. The average AUCs over the SCOP dataset, calculated as described in the Results section, are compared among different methods. Data from the SGM and SSM methods are obtained as published in [14]. These two methods are compared against the fragbag and two topic-based representations (as published here and in [54]).

Figure 6 compares the average AUCs obtained using fragbag and our topic-based representations and additionally places them in a larger context by comparing them to two methods, SSM [28], representative of alignment-based methods, and SGM, representative of filter methods [42]. The average AUCs reported for these methods are obtained as published in [14]. Additionally, average AUCs obtained over topics as reported in [54] with 10 topics are shown. Figure 6 shows that SSM is the best performer, followed closely by fragbag and the rest. LDA and SGM are comparable.

In particular, the average AUCs on each SAS threshold obtained with the fragbag and topic-based representations are listed in Table 1 for a direct comparison. Two observations can be drawn. First, both representations, fragbag and topic-based, are equally effective at capturing structural neighbors at each of the three SAS thresholds. Second, under each representation, the effectiveness is higher at lower SAS thresholds (above 0.8 at a SAS threshold of 2.0Å), allowing us to conclude that the representations have an easier time capturing close homologs than remote homologs. However, performance on remote homologs remains good (higher than 0.7 at a SAS threshold of 5Å ). Taken together, this experiment allows concluding that the topic-based representation allows capturing structural similarity and can be employed to rapidly extract structural neighbors (close and remote homologs) of a given protein with known structure.

**Table 1:** Fragbag/Topic AUCs

	5Å	3Å	2.5Å
Fragbag [29]	0.75	0.77	0.89

Topic-based (Here)	0.72	0.74	0.85

### Automated mapping and organization of protein structure space

We now proceed to demonstrate how the fragbag and topic-based representations can be used to provide low-dimensional maps or categorizations of the known protein structure space.

#### Analysis of fragment-based embeddings of protein structure space

**Figure 7:**
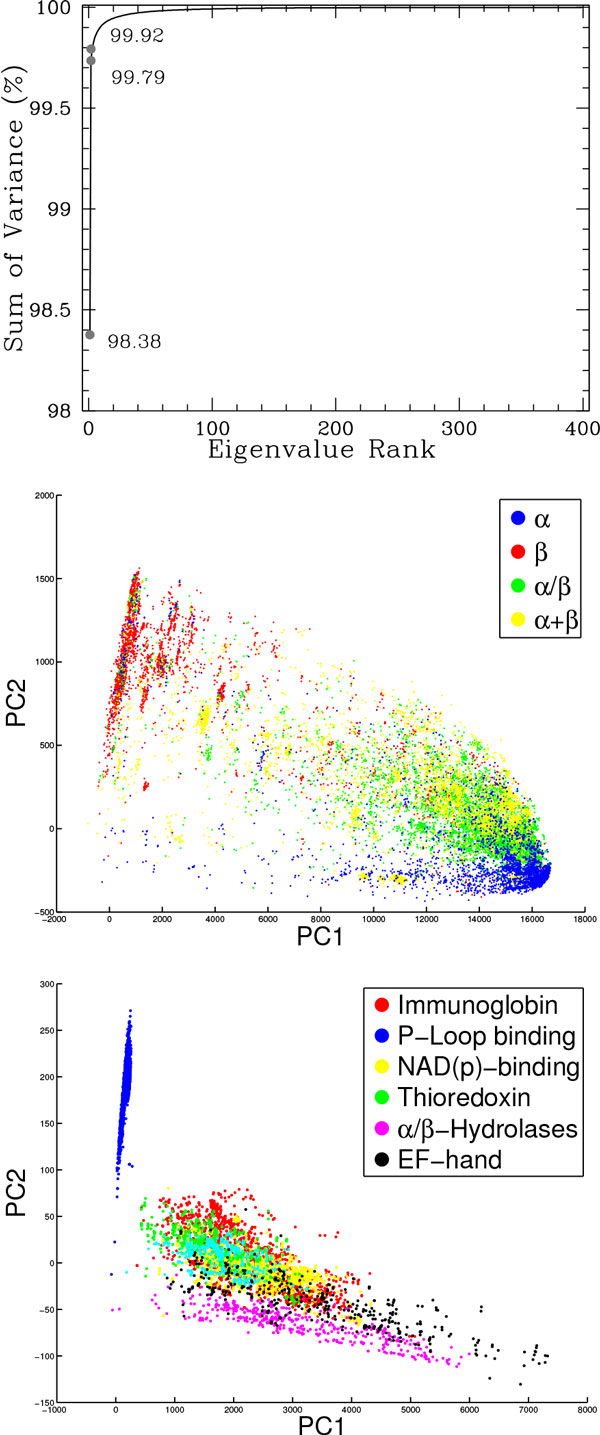
**PCA analysis of SCOP domains**. Top panel shows accumulation of variance from PCA. The top two PCs capture more than 99% of variance. Middle and bottom panels show the projection of SCOP domains on the top two PCs. Different colors are used to separate classes (middle panel) and superfamily (bottom panel).

We conduct a PCA analysis on the SCOP dataset described above. The accumulation of variance on the ordered eigenvalues, plotted in Figure 7 (top panel), shows that the first two PCs capture more than 99% of the variance, demonstrating that projection on these two PCs provides an informative low-dimensional space of the protein structure space. We visualize such a map in Figure 7 (middle and bottom panels). We employ different color-coding schemes to track proteins that belong to the same fold or the same superfamily in SCOP.

Figure 7 (middle panel) shows the highest-populated classes in the first level of the hierarchy; these are, namely, *α, β, α *+ *β*, and α/β proteins. The PCA map in Figure 7 (middle panel) clearly shows that the first PC captures most of the all-*α *proteins, whereas the second PC captures most of the all-*β *proteins. There is more variation in the proteins assigned to the all-*β *class, but a closer inspection reveals some of these proteins contain one or a few *α*-helices (data not shown). As expected, the other two folds, which combine a-helices and β-sheets, span the space. The layout of protein folds in this low-dimensional map is in agreement with other studies [47,34].

Figure 7 (bottom panel) selects six top-populated SCOP superfamilies. Proteins in a superfamily have similar function. In agreement with the study in [34], which pursues a Multi Dimensional Scaling (MDS) mapping of the protein structure space (employing a different parameterization), the two-dimensional map revealed from the PCA analysis shows good functional co-localization of these superfamilies. That is, proteins in the same superfamily are also neighbors in the projected space. This result further illustrates the usefulness of low-dimensional maps that allow visualization of the protein structure space.

It is interesting to note that the fragbag representation essentially unravels the non-linearity in the protein structure space. In other studies, most notably by Kim and colleagues [34], MDS has been central to obtaining an accurate low-dimensional projection of the structure space. The parameterization of a protein structure in that study was not based on a BOW representation.

#### Topics have semantic meaning in the protein structure space

**Figure 8:**
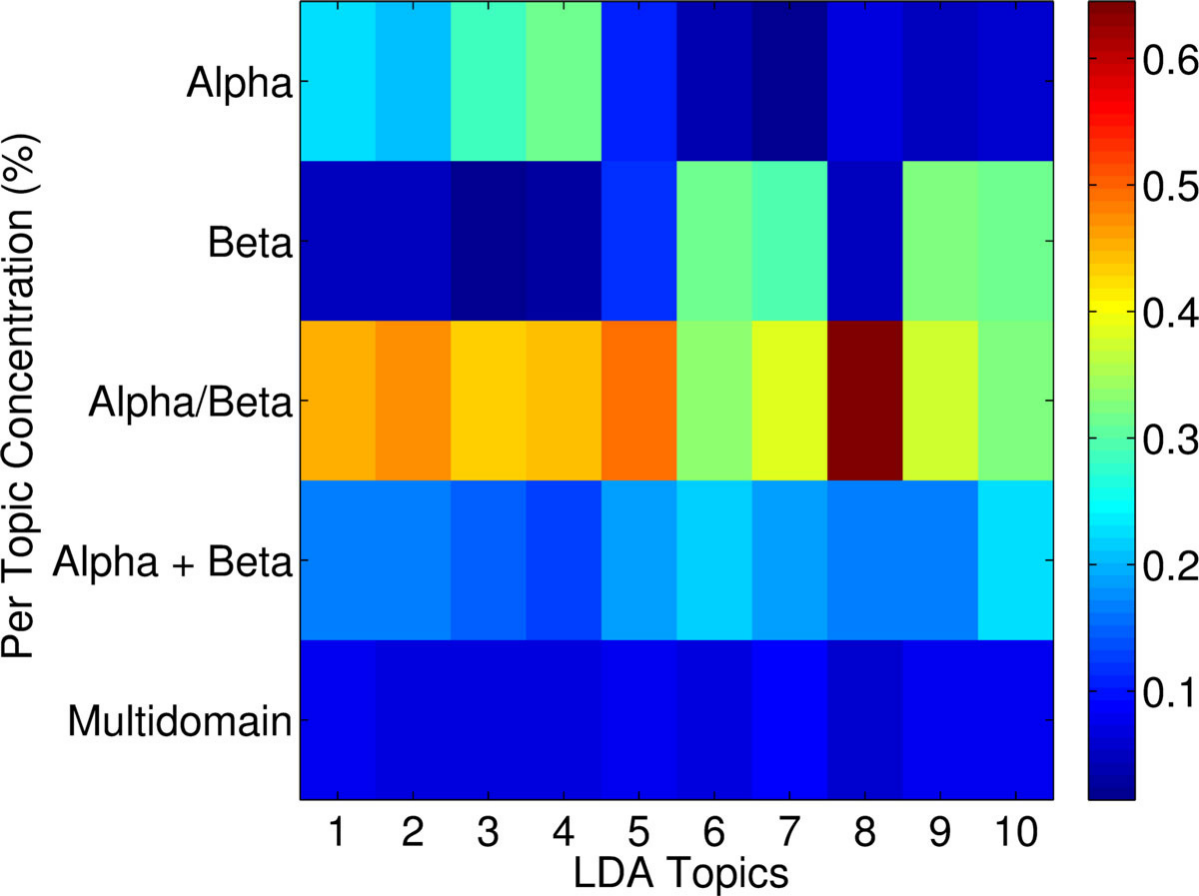
**Heatmap topic analysis on SCOP folds**. Heatmap highlights "signature" topics per class in the fold level of the SCOP hierarchy. Blue-to-red color scheme tracks low-to-high probabilities measured as detailed in Methods.

Taken together, the above analysis suggests that topic space is an informative low-dimensional embedding of the protein structure space that allows capturing structural similarity. To complete the analysis, we elucidate topic signatures per SCOP class at different levels of the SCOP hierarchy. The heatmap shown in Figure 8 color-codes topics per class at the fold level of the SCOP hierarchy in a blue-to-red color scheme tracking low-to-high probabilities measured as detailed in Methods. The results shown in Figure 8 suggest that topics 1-4 are over-represented in the α class but under-represented in the *β* class. This is reversed for topics 5-10. In contrast, the other classes either have a high mixture or a low mixture of each topic. Correlating these results with those shown in Figure 5 provides an explanation for why this is the case. Topics 1-4 are related to *α*-helical topologies, as evidenced by the top fragment shown. Topics 5-10 are related instead to *β*-sheet topologies. Put together, these results demonstrate that classes at the fold level of the SCOP hierarchy have unique topic signatures. It is worth emphasizing that this result is made even stronger when considering that, often, domains assigned to the *β *class may contain a few *α*-helices (data not shown). The analysis suggests that topics capture structural categorization.

**Figure 9:**
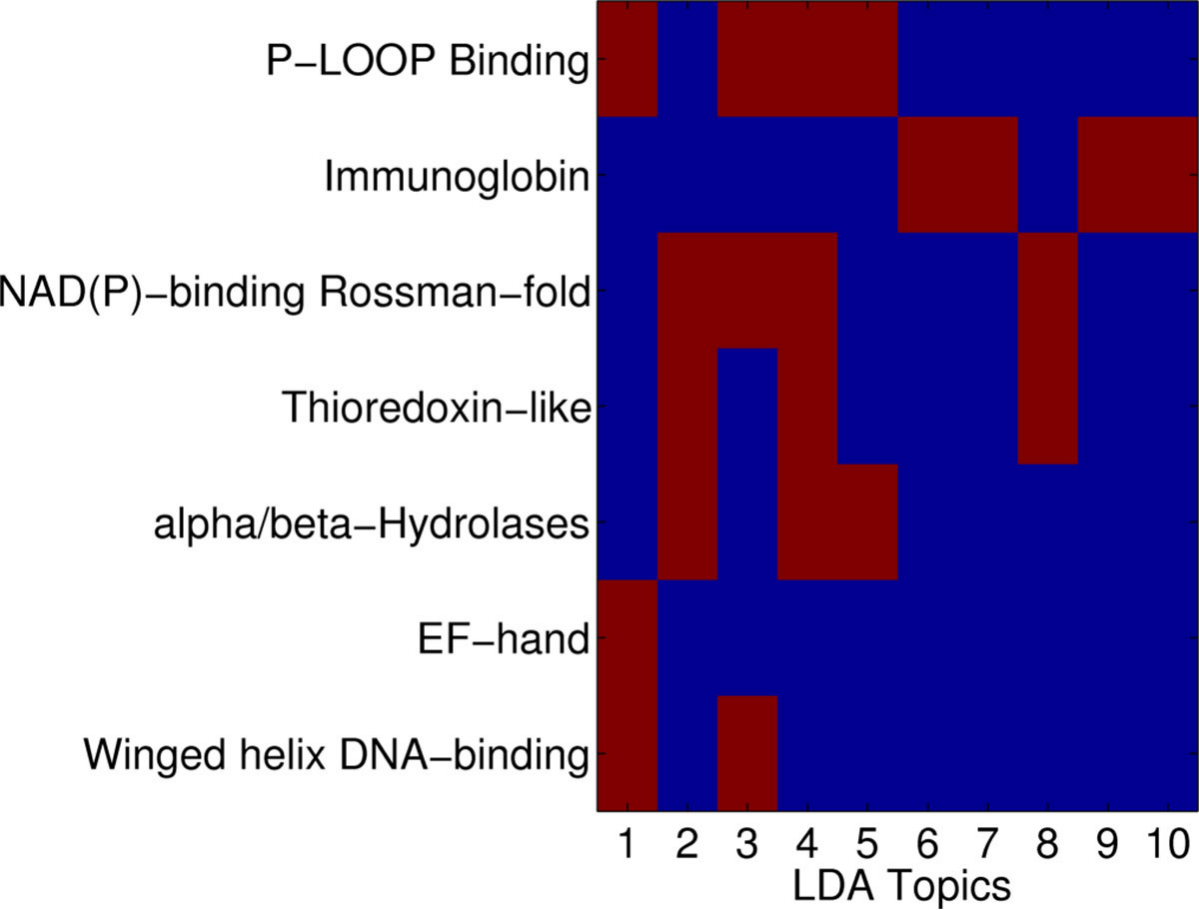
**Heatmap topic analysis on SCOP classes**. Heatmap highlight "signature" topics per class in the superfamily level of the SCOP hierarchy. Blue-to-red color scheme tracks low-to-high probabilities measured as detailed in Methods.

The heatmap in Figure 9 is prepared through the technique detailed in Methods to correct for the high variance in population sizes of top superfamilies in SCOP. Blue indicates low presence of a topic, and red indicates high presence. The results shown in Figure 9 suggest that superfamilies have unique topic signatures. For instance, the immunoglobulin domain has many of topics 5-10 over-represented. This is encouraging, as inspection of these topics in Figure 5 reveals that they are high in *β*-sheets, and immunoglobulin domains are all-*β *proteins. On the other hand, the P-loop Binding domain is rich in *α*-helices. Encouragingly, the topics that are over-represented in this superfamily are topics 1-4, which capture *α*-helical fragments, as shown in Figure 9. The winged helix DNA-binding domain is significantly represented in topics 1 and 3, both having high concentration of *α*-helical fragments. This agrees with the SCOP classification of this domain as all *α*. Similarly, EF-hand is only significantly represented in topic 1, which is dominated by *α*-helical fragments. This is in agreement with the all *α *SCOP classification. The topic signatures capture the other superfamilies, as well, suggesting that topics additionally capture functional categorization.

### Predicting superfamily membership

**Figure 10:**
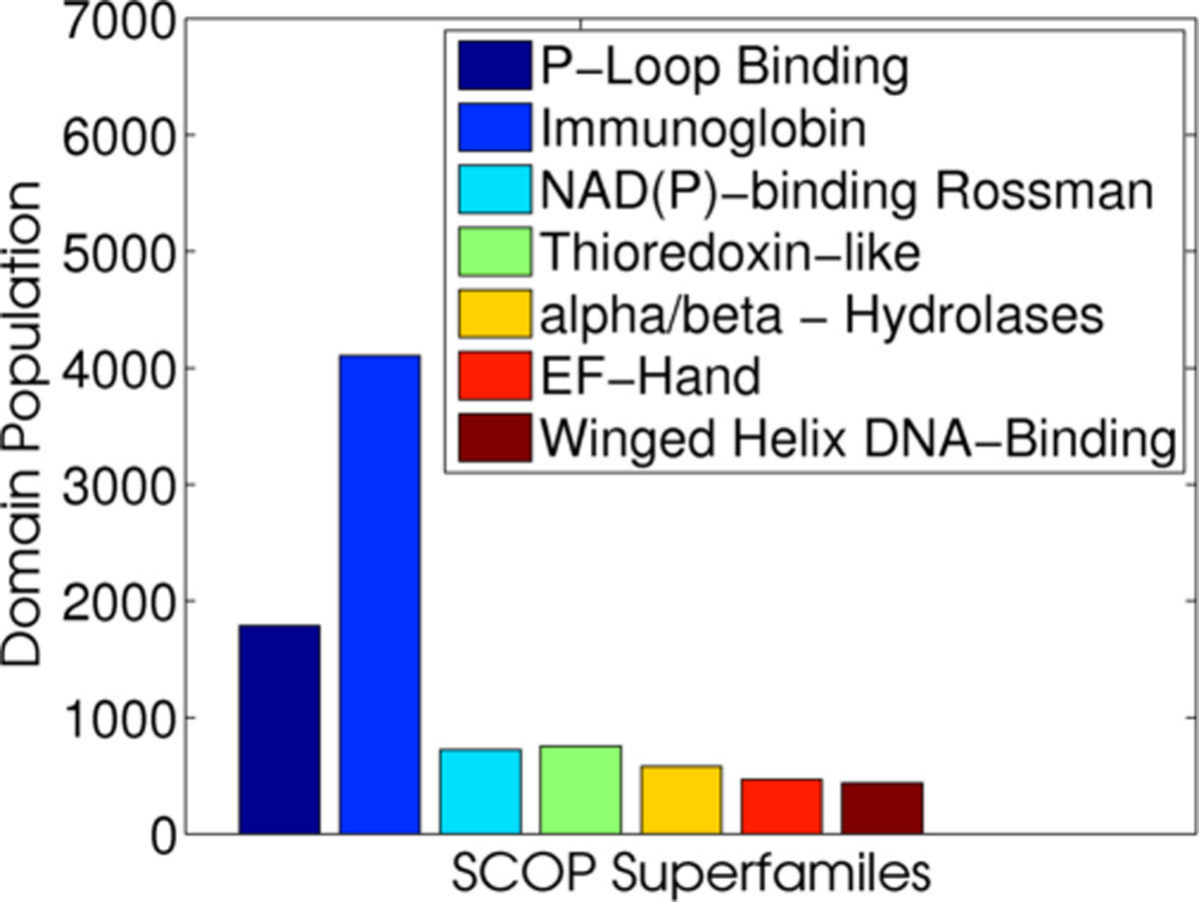
**SCOP superfamily distribution**. The distribution per superfamily is shown for the protein domains in the 7 most-populated superfamilies in SCOP. These domains are treated as training data for SVMs to classify proteins by superfamily.

Finally, a set of 7 classifiers is built as described in the Methods section. This experiment is repeated twice, once using the fragbag and the other using the topic-based representation. The distribution of the protein domains employed as training data in each case across the 7 superfamilies is shown in Figure 10. The performance of each of the 7 SVM classifiers in 10-fold validation is shown in Table 2. Very high accuracy (> 80%), TPR (> 0.8), AUC (> 0.83), and low FPR (< 0.3) are obtained on each superfamily whether using fragbag or the topic-based representation. The fragbag representation allows for slightly better classification performance. These results confirm that the topic-based representation, while only 10-dimensional as compared to the 400-dimensional fragbag representation, can be used to build effective classifiers of proteins, even at the superfamily level of detail.

**Table 2:** SCOP SVM Classification Results.

	Fragbag Representation				Topic-Based Representation			
**SCOP Superfamily**	**Acc. (%)**	**TPR**	**FPR**	**AUC**	**Acc. (%)**	**TPR**	**FPR**	**AUC**

P-Loop Binding	96.4	0.98	0.05	0.95	84.3	0.97	0.29	0.84

Immunoglobin	100.0	1.00	0.00	1.000	99.9	0.99	0.0	1.0

NAD(P)-binding Rossman	98.7	0.99	0.02	0.99	90.9	0.94	0.13	0.91

Thioredoxin-like	98.8	0.98	0.01	0.99	80.2	0.92	0.32	0.80

alpha/beta Hydrolases	99.1	1.00	0.02	0.99	92.7	0.95	0.10	0.93

EF-hand	100.0	1.00	0.00	1.000	98.8	0.99	0.01	0.99

Winged helix DNA-binding	98.7	0.98	0.01	0.99	84.4	0.79	0.11	0.84

Performance is reported for the 7 SVM classifiers identifying a protein domain as being a member of one of the seven SCOP superfamilies. Accuracy (Acc.) is the sum of true positives and true negatives divided by the number of samples. Reported values are rounded up after the second decimal sign.

## Conclusions

In this work we have investigated a novel low-dimensional categorization of protein structure space combining mature and popular tools in text mining with work in structural bioinformatics. The LDA-obtained topic representation of protein structure is analyzed in detail for its ability to summarize a protein structure with multinomial distributions. Our investigation reveals that indeed meaningful topics can be discovered in protein structures, and that these topics can in turn be used to reveal similar protein structures and organize protein structure space.

In particular, results presented in this work suggest that topic-based categorization of protein structures preserves structural and functional co-localization. Specifically, topics obtained through LDA are shown to capture structural similarity with sufficient accuracy on both close and remote homologs and additionally yield a low-dimensional organization of the protein structure space that preserves groupings by structure and function. Topics are also shown to provide sufficient discriminative power to standard supervised learning classifiers like SVMs for predicting superfamily membership. Taken together, the results suggest that the LDA-obtained topic representation of protein structure can be used to aid classification in structural databases.

The work presented in this paper opens exciting new venues in extracting and organizing information about protein structures and protein structure space through mature tools in text mining. We additionally hope that this work can inspire further investigation of higher-order representations of protein structures both for structure comparison and for investigating the relationship between protein sequence, structure, and function. Specifically, future work may choose to further mine and refine the topic-based representation in a way that provides visually-friendly categorizations of protein structure to potentially assist hierarchic organizations in current structural databases, such as SCOP and CATH. Additional future work can explore employment of LDA over structure components others than backbone fragments.

## Competing interests

The authors declare that they have no competing interests.

## Authors' contributions

KM suggested the methods and the performance study in this manuscript and drafted the manuscript. JV helped design and implement the techniques, carried out some of the analysis, and investigated the results. AS and DB guided the study, provided comments and suggestions on the presented methodology and performance evaluation, and improved the manuscript writing.

## Declarations

The publication of this work was funded by NSF CCF Award No. 1016995 and NSF IIS CAREER Award No. 1144106 to AS.

This article has been published as part of *BMC Bioinformatics *Volume 15 Supplement 8, 2014: Selected articles from the Third IEEE International Conference on Computational Advances in Bio and Medical Sciences (ICCABS 2013): Bioinformatics. The full contents of the supplement are available online at http://www.biomedcentral.com/bmcbioinformatics/supplements/15/S8.
